# Origins of increased airway smooth muscle mass in asthma

**DOI:** 10.1186/1741-7015-11-145

**Published:** 2013-06-06

**Authors:** Rachid Berair, Ruth Saunders, Christopher E Brightling

**Affiliations:** 1 Institute for Lung Health, Department of Infection, Immunity and Inflammation, University of Leicester, Leicester LE3 9QP, UK

**Keywords:** Airway remodeling, Airway smooth muscle, Asthma, Fibrocytes, Mesenchymal stem cells

## Abstract

Asthma is characterized by both chronic inflammation and airway remodeling. Remodeling - the structural changes seen in asthmatic airways - is pivotal in the pathogenesis of the disease. Although significant advances have been made recently in understanding the different aspects of airway remodeling, the exact biology governing these changes remains poorly understood. There is broad agreement that, in asthma, increased airway smooth muscle mass, in part due to smooth muscle hyperplasia, is a very significant component of airway remodeling. However, significant debate persists on the origins of these airway smooth muscle cells. In this review article we will explore the natural history of airway remodeling in asthma and we will discuss the possible contribution of progenitors, stem cells and epithelial cells in mesenchymal cell changes, namely airway smooth muscle hyperplasia seen in the asthmatic airways.

## Introduction

The immunopathological features of asthma include chronic airway inflammation and airway remodeling [1]. Over the past few decades, the research into the pathophysiology and treatment of asthma has been primarily focused on the inflammatory side of the disease with most new therapies targeting various aspects of inflammation. Despite this, there persists a huge unmet need, especially for patients with severe asthma who have persistent debilitating symptoms and increased morbidity and mortality despite maximum anti-inflammatory therapy. Severe asthmatics account for 5 to 10% of asthma sufferers and are responsible for more than 50% of asthma related costs [2,3].

Airway remodeling, a collective term describing the wide range of histopathological structural changes seen in the asthmatic airway wall, has been recognized for almost a century; however, significant gaps still remain in our understanding of various aspects, including its natural history, etiology, molecular and cellular basis and, more importantly, its clinical and physiological relevance. Airway remodeling is characterized by increased airway smooth muscle (ASM) mass, sub-epithelial fibrosis, goblet cell hyperplasia, sub-mucosal mucus gland hypertrophy, neoangiogenesis, an abnormally fragile epithelium and an increased number of activated fibroblasts and myofibroblasts with subsequent increased deposition of extracellular matrix proteins [4-7]. Although most of the aforementioned characteristics of airway remodeling contribute to the airway narrowing seen in asthma, increased ASM mass, due to both ASM hyperplasia and hypertrophy, and sub-epithelial fibrosis, are the most critical. ASM mass is a major component responsible for bronchoconstriction of airways in response to stimuli and, together with sub-epithelial myofibroblast hyperplasia, is the major determinant of persistent airflow obstruction [8,9]. Myofibroblasts are contractile mesenchymal cells located in the submucosa between the airway smooth muscle bundle and the epithelium. In addition to secreting extracellular matrix proteins, myofibroblasts have similar characteristics and function to ASM and probably represent part of the spectrum of plasticity of mesenchymal cells (fibroblasts ⇔ myofibroblasts ⇔ ASM).

In this review we will attempt to explore the underlying mechanisms leading to increased ASM mass in the asthmatic airways and the possible implications in developing new asthma therapies.

### What is the stimulus and natural history of airway remodelling?

Before attempting to unfold the origins of increased ASM mass in asthma it is important to try and understand the natural history of airway remodeling, including its initiation, its progress and, importantly, its relation to airway inflammation. The airway is exposed to a number of potential stimuli including pathogens, pollutants and allergens. The role of these stimuli in the genesis of airway remodeling, particularly with regard to ASM mass, is poorly understood. In an allergen challenge study of asthmatics who had bronchial biopsies pre-, and one and seven days post-challenge, sub-epithelial fibroblast number and activation, and reticular basement membrane (RBM) thickening, increased and persisted over the seven days and was associated with increased airway hyper-responsiveness. In contrast, inflammation observed at day one had resolved by day seven [10]. This suggests that remodeling occurs in response to allergen challenge; is related to airway dysfunction, might require inflammation for its initiation, but is not dependent upon persistent inflammation.

Viruses are the most common cause of asthma exacerbations and are, therefore, possibly involved in the induction of remodeling although the evidence is scanty. Rhinovirus *in vitro* can promote the release of matrix proteins from cultured ASM suggesting it may influence remodeling [11]. Another surprising factor that influences remodeling is the pure mechanical force that is generated by constriction of airways. Bronchoconstriction induced by using the indirect ASM spasmogen methacholine, was found to promote RBM thickening [12]. However, whether any of these stimuli affect ASM mass is unknown.

The natural history of airway remodeling is unknown. Whether a single or a few airway insults over weeks to months, versus several repeated insults over many years, are required to cause airway remodeling is unclear. What is clear, interestingly, is that changes in RBM thickness and ASM mass occur early in the natural history of asthma. Increased RBM thickness is present in preschool children with wheeze, and only those who also show signs of airway remodeling are subsequently diagnosed with asthma at school age [13,14].

The dynamic relationship between inflammation and remodeling is poorly understood. The classical paradigm was that repeated inflammation of the airways, mainly due to exaggerated T-helper type-2 immune response, leads to defective repair and remodeling. However, there is increasing evidence discrediting this simple concept. In children with established severe disease, RBM thickening and increased ASM mass are present and dissociated from inflammation [15]. Similarly, Cokugras *et al*. reported in 10 children with moderate asthma evidence of remodeling preceding inflammation [16]. All these observations raise important questions about the timescales required for the establishment of airway remodeling and the timing of interventions to alter the natural history of the disease.

### What are the origins of increased ASM mass in asthma?

In humans, ASM is a normal component of both large and small airways. The exact physiological function of ASM has always been a controversial issue with some claiming that it plays a role in maintaining bronchial tone and lung ventilation, while others consider it a vestigial ruminant with no function akin to the intestinal appendix [17].

**Figure 1:**
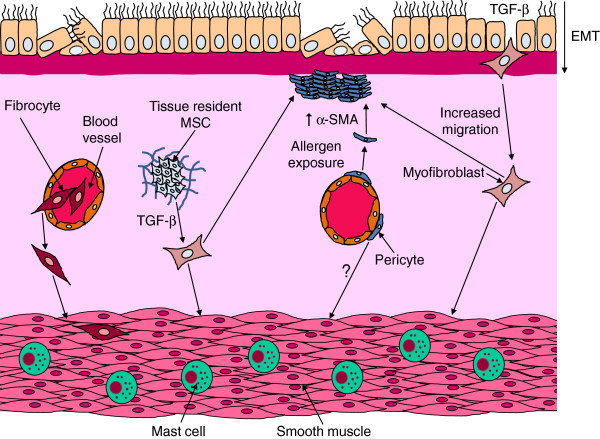
**The potential origins of myofibroblast and ASM hyperplasia in asthmatic airways.** Increased airway smooth muscle (ASM) and myofibroblast cell numbers could result from recruitment of fibrocytes from the peripheral circulation to the airway tissue; or from differentiation of tissue resident mesenchymal stem cells (MSCs); or from epithelial-mesenchymal transition (EMT) of epithelial cells. Additionally, pericytes may also contribute to this process. To transform to functioning ASM or myofibroblasts, all these cells lose their non-mesenchymal markers and acquire mesenchymal characteristics like increased *α*-SMA expression.

The increase in ASM volume in asthma is caused by both hyperplasia and hypertrophy [8,18]. Although there is general agreement that ASM hyperplasia seems to be more dominant and significant, its origin remains uncertain. These new ASM cells might be a consequence of increased proliferation and/or prolonged survival of pre-existing ASM; or could result from recruitment of non-ASM cells that would transform and differentiate into ASM. These potential ASM progenitors include true multipotent mesenchymal progenitor and stem cells, either located within airway tissues or derived from peripheral blood, and airway epithelial cells which can migrate and transform into a mesenchymal cell phenotype through a biological process called epithelial-mesenchymal transition (EMT) (Figure 1).

#### ASM proliferation and survival

In biology, hyperplasia of any tissue is usually caused by the increased rate of division of primary cells constituting the tissue, that is, proliferation. Consequently, proliferation has initially been regarded as the main mechanism underlying ASM hyperplasia in asthmatics. However, despite extensive research, the evidence for ASM proliferation in asthmatics remains largely inconclusive. In some *ex vivo* studies [19,20], but not others [21,22], asthmatic ASM showed increased proliferation compared to non-asthmatics. More importantly, several *in vivo* studies have failed to demonstrate proliferation [8,23]. Similarly, there is a lack of evidence to suggest that *ex vivo* ASM from asthmatics has an altered rate of survival. It therefore remains unclear whether altered cell proliferation or survival contributes significantly to ASM hyperplasia in asthmatics, although, in our opinion, this seems unlikely to be the principal underlying mechanism.

#### Peripheral blood-derived mesenchymal progenitors

Fibrocytes are the only blood-derived mesenchymal progenitors identified as a possible source of ASM in asthma. They were first described by Bucala *et al*. in 1994 in a mouse model of wound healing, as a distinctive group of cells with both hematopoietic and mesenchymal properties [24]. Since then fibrocytes have gained increasing prominence with emerging evidence of their involvement in the aberrant tissue repair evident in a number of fibrotic lung disorders, including pulmonary hypertension, idiopathic pulmonary fibrosis and asthma [25]. Fibrocytes normally constitute 1% of the peripheral blood leukocytes and express a wide range of hematopoietic stem cell, myeloid and mesenchymal markers, including CD34, CD11b, CD13, CD45, CXCR4, CCR7, procollagen-I, vimentin and *α*-smooth muscle actin [26]. After leaving the circulation and once in any tissue, fibrocytes lose the expression of their hematopoietic markers and gain mesenchymal markers accentuated by transforming growth factor-*β* (TGF-*β*) and endothelin-1, both of which are found at increased levels in asthmatic airway tissues compared to non-asthmatic tissue [27,28].

The first evidence of fibrocyte involvement in asthma was described a decade ago. Schmidt and associates showed, in a group of allergic asthmatics, an increase in the number of cells co-expressing CD34, procollagen-I mRNA, and *α*-smooth muscle actin in the sub-epithelium following allergen exposure [29]. The authors also showed, by labeling and tracking fibrocytes in an experimental mouse model of allergic asthma, that fibrocytes are recruited from the circulation after allergen exposure. In a study connecting fibrocytes directly to remodeling, Nihlberg *et al*. showed a clear correlation between the thickness of the basement membrane and the number of tissue fibrocytes in a small group of patients with steroid-naïve mild asthma [30]. Wang *et al*. demonstrated a relationship between peripheral blood fibrocyte number and airflow obstruction [31]. The increased proliferation of fibrocytes in asthmatics with chronic airflow obstruction has recently been linked to oxidative stress mediated via up-regulation of the epidermal growth factor receptor pathway [32]. The clearest evidence of the direct contribution of fibrocytes to ASM hyperplasia *in vivo* was presented by Saunders *et al*. [33]. They showed that, compared to normal controls, severe refractory asthmatics have an increased number of fibrocytes in the peripheral circulation and also in the airway submucosa. More interestingly, they demonstrated an increased number of fibrocytes in the ASM bundle in asthmatics of all severities. It is important to note that despite the relatively large number of subjects in this study (51 asthmatics and 33 healthy controls), the researchers failed to demonstrate any link between fibrocytes and lung function.

Although the homing of fibrocytes to damaged tissues is not fully understood, a few chemotactic pathways have been identified and others suggested. Human fibrocytes express several chemokine receptors, including CCR2, CCR3, CCR5, CCR7 and CXCR4 [34]. In particular, CXCR4 and its ligand, CXCL12, were proven to play an important role in fibrocyte homing to the lung in a model of bleomycin-induced pulmonary fibrosis [35]. IL-33, which is increased in asthmatic airways and is related to severity, has been shown to have a strong chemotactic and proliferative effect on asthmatic fibrocytes compared to healthy controls [36]. Given the epithelial origin of IL-33, IL-33 might be important for recruitment of fibrocytes from the circulation and subsequent sub-epithelial myofibroblast accumulation but not specifically for fibrocyte migration to the ASM bundle within the airway tissue. *In vitro*, platelet-derived growth factor originating from ASM, and CCL19 released by mast cells within the ASM-bundle, can promote fibrocyte and myofibroblast migration, respectively, towards the ASM [33,37].

#### Mesenchymal stem cells

Our knowledge and understanding of the role of mesenchymal stem cells (MSCs) in general remains relatively limited. The research into the subject has been hindered by many factors including the lack of a universally agreed upon definition and the relative paucity of identifiable specific cell markers. MSCs lack hematopoietic and epithelial markers and possess multiple other markers suggestive of multi-lineage mesenchymal differentiation potential.

Tissue resident MSCs are an essential part of any connective tissue and have important biological functions in healing, repair and regeneration [38]. MSCs exist in most tissues, including the bone marrow (also known as bone marrow stromal cells or BMSC). BMSC have recently generated much excitement and attention due to their anti-inflammatory and immunomodulatory action. *In vivo*, BMSC have clearly been shown to improve inflammation and, *in vitro*, BMSC were also proven to inhibit most inflammatory cells [39]. Treatment with BMSC has resulted in promising results in steroid-resistant graft versus host disease and also in disease models of inflammatory diseases [40]. With regard to experimental use of BMSC in asthma, this has been promising although only limited to animal and *in vitro* studies [41]. Mainly in murine models of asthma, treatment with BMSC reduced inflammation and airway remodeling [42,43].

In the lung, however, the role of resident MSCs is controversial. There is some evidence to support the involvement of lung MSCs in the pathogenesis of some fibrotic lung conditions. Studies have shown that MSCs undergo transformation into a myofibroblastic phenotype in premature infants, possibly contributing to the pathogenesis of bronchopulmonary dysplasia, and in allograft lungs, possibly assisting in the development of bronchiolitis obliterans [44,45]. This suggests that lung resident MSCs can potentially transform into a smooth muscle phenotype and possibly contribute to ASM hyperplasia in asthma although the evidence is lacking. Bentley *et al*. showed increased numbers of MSCs in the lung of an ovalbumin-sensitized mouse model following aerosol challenge, although this was not linked to any of the features of remodeling [46].

Pericytes are contractile cells normally wrapped around the endothelium of most microvasculature. They have been found to fulfill both the multilineage and markers criteria for MSCs and many nowadays argue that pericytes could be the source of all MSCs. In kidney injury, pericytes have been found to be a source of mesenchymal contractile cells. Interestingly, pericytes might be involved in ASM hyperplasia in asthma, although the evidence is limited to only one report in the literature. In a murine asthma model, upon aeroallergen exposure, pericytes seem to detach from vasculature and migrate to the sub-epithelium of the airway, with up-regulation of α-smooth muscle actin [40].

To conclude, limited evidence seems to suggest that, unlike BMSC, lung resident MSCs lack beneficial immunomodulatory action and can even contribute to disease pathogenesis. The cause of this difference and whether lung resident MSCs contribute to the pathogenesis of remodeling in asthma remain to be answered.

#### Epithelial-mesenchymal transition

Epithelial-mesenchymal transition is a biological process in which epithelial cells lose adhesion and acquire mesenchymal traits, including increased motility and migration, prolonged survival and enhanced extracellular matrix proteins production. EMT, mediated mainly by the action of TGF-*β,* is known to play a role in cell differentiation during embryogenesis and also in cancer proliferation and metastasis [47]. Moreover, EMT has also been shown to contribute to the pathophysiology of a number of fibrotic diseases, including idiopathic pulmonary fibrosis.

The airway epithelium in asthma is fragile with reduced cell to cell adhesion possibly making EMT more likely [48]. However, despite this, the evidence for the significance of EMT in asthma remains weak. Heijink *et al*. reported EMT in TGF-*β*–primed cultured human bronchial epithelium when exposed to aeroallergens [49]. In a murine model of asthma, Johnson *et al*. demonstrated, through labeling of epithelial cells, clear evidence of EMT *in vivo* after prolonged allergen exposure [50]. Finally, Hackett and colleagues compared the effect of TGF-*β* on cultured bronchial epithelium from eight asthmatics and nine non-asthmatics [51]. They showed evidence of EMT throughout the epithelium in the asthmatic group, while in the non-asthmatics the changes were limited to the basal layer of cells. The lack of *in vivo* data on EMT in asthma makes quantifying the relative contribution of EMT to mesenchymal cell population expansion very difficult.

## Conclusion

The factors governing ASM and myofibroblast hyperplasia in asthma are still not fully understood. Further human studies following experimental challenges or novel interventions, that impact upon airway remodeling, such as thermoplasty, are required. Thermoplasty is a newly licensed therapy for severe asthma that applies thermal energy to the airway wall via a bronchoscope. In early animal studies and in humans receiving thermoplasty prior to lung resection for cancer, ASM mass is reduced, suggesting that the improved exacerbation frequency and health status following therapy in asthma may be in part due to a reduction in ASM mass [52]. Importantly, to date this has not been confirmed in asthma. How the airway responds to this thermal injury might shed light not only upon the mechanism of action of this therapy, but also provide insights into airway repair in response to injury. Such studies will help to determine the relationship between these structural changes and disordered airway function. As discussed, the role of mesenchymal stem and progenitor cells in airway smooth muscle remodeling in asthma is obscure. There is a significant possibility that a degree of heterogeneity exists among these cells with some having immunomodulatory properties that ought to be promoted and others having pro-remodeling properties that ought to be suppressed, although this remains to be proven. In conclusion, the last few decades have advanced greatly our understanding of the role of inflammation in asthma. The challenge for the next decade is to understand the mechanisms driving airway remodeling, particularly increased ASM mass and its clinical relevance, to inform the development of new therapies.

## Abbreviations

ASM: Airway smooth muscle; BMSC: Bone marrow stromal cells; EMT: Epithelial-mesenchymal transition; IL: Interleukin; MSCs: Mesenchymal stem cells; RBM: Reticular basement membrane; TGF-β: Transforming growth factor-*beta*.

## Competing interests

The authors declare that they have no competing interests.

## Authors' contributions

RB reviewed the literature and prepared the draft manuscript. RS contributed to the content of the article, especially the section on fibrocytes. RS also drafted the accompanying figure. CEB conceived the design and structure of the article and had the final decision to submit the article for publication. All authors contributed to the writing of the manuscript and have approved the final version for submission.

## Authors' information

RB is a clinical research fellow in the Department of Infection, Immunity and Inflammation at the University of Leicester. He is also a part-time PhD student in the same department and his future thesis will be focused on the relation between remodeling and function in the asthmatic airways. RS is a Postdoctoral Research Associate in the Department of Infection, Immunity and Inflammation at the University of Leicester. Her special interests include the mechanisms involved in airway remodeling in asthma. CEB is a Wellcome Senior Research Fellow and Clinical Professor in Respiratory Medicine. His research in focused on the immunopathogenesis of airways disease, including asthma and COPD.

## Pre-publication history

The pre-publication history for this paper can be accessed here:

http://www.biomedcentral.com/1741-7015/11/145/prepub
